# Efficacy and safety of dual-targeted therapy for refractory inflammatory bowel disease: a retrospective case series from three tertiary general hospitals in China

**DOI:** 10.3389/fmed.2025.1566828

**Published:** 2025-05-12

**Authors:** Xiaoying Wang, Ye Fang, Jiakai Luo, Yongli Ye, Shuyan Li, Dingting Xu, Xiaoqi Zhang, Yi Jiang, Qiao Yu, Yan Chen

**Affiliations:** ^1^Department of Gastroenterology, The Second Affiliated Hospital, Zhejiang University School of Medicine, Hangzhou, Zhejiang, China; ^2^Department of Gastroenterology, The Second Affiliated Hospital of Wenzhou Medical University, Wenzhou, Zhejiang, China; ^3^Endoscopy Center, The Second Affiliated Hospital, Zhejiang University School of Medicine, Hangzhou, Zhejiang, China; ^4^Nursing Department, The Second Affiliated Hospital, Zhejiang University School of Medicine, Hangzhou, Zhejiang, China; ^5^Department of Gastroenterology, Nanjing Drum Tower Hospital, The Affiliated Hospital of the Medical School at Nanjing University, Nanjing, China

**Keywords:** dual-targeted therapy, combination therapy, refractory inflammatory bowel disease, endoscopic response, clinical response

## Abstract

**Aim:**

Dual-targeted therapy (DTT) may offer a promising approach for treating refractory inflammatory bowel disease (IBD). The aim of this case series was to evaluate the safety and clinical response of DTT in clinical practice.

**Methods:**

We retrospectively analyzed data from refractory inflammatory bowel disease (IBD) patients receiving dual-target therapy (DTT) across several Chinese IBD centers. The treatment combinations included biologic agents (infliximab (IFX), adalimumab (ADA), vedolizumab (VDZ), and ustekinumab (UST) and oral small molecule tofacitinib (TOF). We collected baseline characteristics, clinical and endoscopic activity indices, inflammatory markers (C-reactive protein and albumin), and adverse events to evaluate the clinical effectiveness, endoscopic response, biochemical remission, and safety profile of DTT.

**Results:**

A total of 8 patients with ulcerative colitis (UC) and 10 with Crohn’s disease (CD) underwent DTT at three tertiary hospitals in China. All corticosteroids initiated at baseline (six cases) were completely discontinued within 3 months. Clinical response rates were 88.23% (15/17), 91.67% (11/12), and 100% (7/7) at 3, 6, and 9 months, respectively. Endoscopic response was achieved in 88.89% (8/9) of patients who were evaluated at 9 months. Adverse events included ustekinumab-associated arthralgia and alopecia in one UC patient and tofacitinib-related allergic purpura in another, both of which were subsequently transitioned to monotherapy. Two CD patients developed infections (*Clostridium difficile* and bacterial intestinal infection) at 3 months, were treated with oral antibiotics, and successfully continued their original DTT regimens.

**Conclusion:**

Our findings suggest that dual-target therapy demonstrates promising efficacy and an acceptable safety profile in refractory IBD patients. DTT may represent a valuable therapeutic option for patients who have not responded to conventional monotherapies.

## Introduction

Inflammatory bowel disease (IBD) encompasses chronic, recurrent inflammatory conditions of the gastrointestinal tract, consisting of ulcerative colitis (UC) and Crohn’s disease (CD). In recent years, the widespread clinical adoption of various biologic agents and small-molecule drugs has advanced IBD management (1, 2). Despite these advances, clinical outcomes remain suboptimal, with only 30–50% of patients achieving remission in the first year of treatment (3–6), and a significant proportion still requiring surgical intervention (7).

This therapeutic challenge arises in part from the absence of validated predictive tools to assess individual drug sensitivity, resulting in disease progression despite prompt intervention. Indeed, contrary to all expectations, studies indicate that IBD-related hospitalization rates have not decreased significantly in the biologic agents era (8). This persistent unmet need has led to the concept of a “monotherapy ceiling” in IBD treatment (9), which is particularly evident in patients with extraintestinal manifestations or concurrent autoimmune conditions.

Given that IBD pathogenesis involves multiple inflammatory pathways, simultaneously targeting different mechanisms may enhance therapeutic efficacy. Randomized controlled trials have already demonstrated that combining infliximab with an immunosuppressant significantly outperforms monotherapy (10). More recently, novel biologic agents such as vedolizumab and ustekinumab—offering improved selectivity and safety profiles compared to traditional immunosuppressants—have opened new opportunities for combination therapy approaches.

Dual-targeted therapy (DTT)—combining two biologic agents or a biologic with a small molecule drug—represents a promising strategy to overcome the limitations of monotherapy (11). Despite promising safety and efficacy data from preclinical case series with DTT, a subset of patients still failed to achieve clinical remission (11–16). The recent VEGA and EXPLORER studies further support the potential superiority of combination therapy over individual monotherapies (17, 18). However, robust data on the efficacy and safety of DTT in Asian populations remain notably limited. Therefore, we analyzed outcomes from three major IBD centers to evaluate the safety and efficacy of DTT in Chinese patients, providing essential insights into this promising therapeutic approach in this population.

## Methods

### Study design and participants

Data were reviewed from patient records at The Second Affiliated Hospital of Zhejiang University School of Medicine, the Affiliated Drum Tower Hospital of Nanjing University School of Medicine, and the Second Affiliated Hospital of Wenzhou Medical College. Approximately 11,400 IBD patients were followed across the three IBD centers. From September 2021 to January 2024, we collected the clinical information on 18 patients with refractory IBD who received DTT via the electronic medical record system. Patients had previously received immunosuppressants [azathioprine (AZA), methotrexate (MTX), tacrolimus (TAC) or cyclosporine A (CSA), tofacitinib (TOF), thalidomide (Thd), or/and biologic agents such as infliximab (IFX), adalimumab (ADA), or vedolizumab (VDZ)]. At the same time, these biologic agents or immunosuppressants had undergone a standardized optimization process. All patients were naïve to the second biologic added. The activity of IBD was considered in combination with clinical presentation, laboratory data, and imaging or endoscopic evaluation. This study was approved by the Institutional Review Board of the Ethics Committee of the Second Affiliated Hospital, School of Medicine, Zhejiang University in China (approval no. 2022–0452). In all cases, informed written consent was obtained from participants or their legal surrogates before enrollment.

Dual-targeted therapy was initiated for patients meeting any of the following criteria: (a) refractory IBD (failing biologic agents and small molecules with ≥2 different mechanisms of action, post-operative CD recurrence after multiple bowel resections, antibiotic-resistant pouchitis, complex perianal CD, or psychosocial barriers to effective management) (19); (b) IBD with active extraintestinal manifestations; or (c) IBD with concurrently active immune-mediated inflammatory disease.

Exclusion criteria were: (1) age <14 or >80 years, (2) pregnancy, (3) malignancies, (4) severe psychiatric disorders, (5) infectious diseases, (6) severe comorbidities (including intestinal perforation or obstruction), and (7) contraindications to biologic therapy.

### Data collection

The Montreal classification was used to describe the extent and behavior of the disease. The clinical activity score for the disease was based on the Harvey–Bradshaw Index (HBI) score for CD and the Partial Mayo Score for UC. Laboratory parameters, including C-reactive protein (CRP), albumin (ALB), and hematocrit were also assessed. Data were collected at 0, 3 months (+/−2w), 6 months (+/−1 m), and 12 months (+/−1 m). Information gathered included baseline characteristics of the patients, aspects of the disease (whether combined autoimmune-related disease), medication use (primary and secondary resistance), surgical history, post-combination therapy, clinical or/and endoscopic data, adverse effects (AEs), and medication intervals (20).

### Outcome definitions

The clinical response was defined for UC as a composite of (a) a partial Mayo score reduction of 3 or more, accompanied by a decrease of at least 30% from baseline and (b) a bleeding subscore reduction of 1 or more from baseline or a bleeding subscore of ≤1. The clinical response in CD was defined as the combined result of the following: a HBI score reduction of 3 or more. For patients with an HBI of ≤4, clinical response was determined by meeting at least one of the following two conditions: (1) CRP normalization and (2) the Limberg score reduction of 2 or more. Endoscopic activity was scored by the Simplified Endoscopic Score-Crohn’s disease (SES-CD) for CD and the Mayo Score for UC. Endoscopic response was defined as a reduction in SES-CD score of >50% for CD patients and a decrease in Mayo Endoscopic Subscore (MES) of ≥1 point from baseline for UC patients (21, 22).

### Statistical analysis

Categorical variables were presented as counts and percentages, and continuous variables were described with medians and interquartile ranges (Q1–Q3). Comparisons of categorical variables were performed using the chi-square test or Fisher’s exact test. All *p*-values were two-sided, with a significance threshold of 0.05.

## Results

### Characteristics of the population

Essential characteristics of 18 patients with uncontrolled refractory Chinese IBD (8 with UC and 10 with CD) are described in Tables 1, 2. The mean age at diagnosis was 22.78 years, the mean disease duration was 9.18 years, and the median follow-up time before DTT was 74 months (22–230 months). Of the 18 participants, 11 (61.11%) were female and 7 (38.89%) were male. There were seven CD cases (70%) with a history of abdominal surgery associated with CD (three ileal surgery alone and four ileal + colon). One of these abdominal surgery patients (10%) had a history of perianal surgery. Additionally, six patients (33.33%) had comorbid inactive autoimmune diseases, and all 18 patients received DTT due to active intestinal inflammation. Fourteen patients had previously received two or more biologic agents and immunosuppressive medications prior to DTT initiation. Three patients had been treated with a single biologic agent, while one patient had received only one immunosuppressive agent. The therapeutic agents were adequately optimized. The mean number of failed biologic agents was 1.38, and the mean number of intolerant or ineffective immunosuppressive agents, intolerant immunomodulators (thalidomide), or oral small molecules (tofacitinib) was 1.17.

**Table 1:** Baseline characteristics.

Characteristics	UC (*n* = 8)	CD (*n* = 10)
Sex (F/M)	7/1	4/6
The median age of diagnosis in years (year)	22.5(20 ~ 42)	23.5(14 ~ 67)
Disease duration (year)	6.2(1.8–19.2)	9(4.5–17.5)
Prior surgery for IBD (non-perianal)	0	7
Perianal surgery	0	3
Autoimmune diseases	3	3
Prior biologic exposures		
IFX (primary non-response/secondary loss of response/side effects)	5/0/1	0/6/3
ADA (primary non-response/secondary loss of response/side affections)	1/1/0	2/1/0
VDZ	5	5
Prior immunomodulator exposures		
AZA/MTX/TAC/Thd/CSA	3/1/2/1/1	5/2/3/1
Prior small molecule exposures		
TOF	2	1
Corticosteroids at baseline	3	3
Immunomodulator at baseline	0	1
Thd at baseline	0	1

IFX, infliximab; ADA, adalimumab; VDZ, vedolizumab; AZA, azathioprine; MTX, methotrexate; TOF, tofacitinib; Thd, thalidomide; TAC, tacrolimus; CSA, cyclosporine A.

**Table 2:** Clinical data at baseline.

Patients	Montreal classification	Clinical disease activity	Endoscopic score^†^	CRP (mg/L)	Autoimmune diseases	Previous biologic agents and immunomodulators	Steroid therapy
UC1	E3	Moderate	2	9.2	Allergic Purpura and Urticaria	IFX, VDZ, AZA, MTX, UST, Thd	Yes
UC2	E3	Moderate	3	3.1	Ankylosing Spondylitis	IFX, ADA, TAC, TOF	Yes
UC3	E3	Moderate	2	0.6	No	IFX, CSA	No
UC4	E3	Moderate	3	7	Allergic Purpura	VDZ, IFX, AZA	No
UC5	E3	Severe	3	0.3	No	VDZ, IFX, TOF	Yes
UC6	E3	Severe	3	13.88	No	AZA	Yes
UC7	E3	Moderate	3	56.24	No	VDZ	No
UC8	E3	Severe	3	6.29	No	VDZ, IFX, ADA	No
CD1	L3B2p	HBI moderate	38	20.7	No	IFX, ADA, AZA, TOF, Thd	Yes
CD2	L3B2	HBI remission	13	7.5	Guillain Barre Syndrome	MTX, Thd	No
CD3	L3B2	HBI remission	NA	10.19	No	IFX, VDZ, AZA, Thd	No
CD4	L3B2	HBI mild	33	31.7	No	IFX, ADA, MTX	Yes
CD5	L3B2	HBI remission	NA	3.08	No	IFX, ADA, AZA, MTX, Thd	No
CD6	L3B3p	HBI remission	9	63.8	No	IFX	No
CD7	L3B2	HBI remission	NA	9.74	No	IFX, VDZ, Thd	No
CD8	L3B2	HBI remission	NA	0.6	Arthralgia	IFX, ADA, TAC	No
CD9	L3B2p	HBI mild	NA	110.35	No	IFX	No
CD10	L3B3p	HBI remission	7	1.9	Ankylosing Spondylitis	IFX, AZA	No

The Montreal classification is used to describe the extent and behavior of the disease. E2: left-sided UC, E3: extensive UC, L3: ileocolonic, L4: upper (can be added), B2: structuring, B3: penetrating, p: perianal; NA: not applicable disease. HBI: Harvey–Bradshaw Index. ^†^Endoscopic score: Mayo score for UC; SES-CD score for CD.

### Implementation and status of dual-target therapy

Patients’ assessments during follow-up are reported in Table 3 (12 maintained on DTT) and Table 4 (6 discontinued DTT). Concomitant corticosteroids were applied at DTT baseline in six cases (four UC and two CD), and all corticosteroids were withdrawn entirely within 3 months. The frequency of drugs used in 18 DTT cases was 15 UST, 10 VDZ, 5 TOF, 3 IFX, and 3 ADA, while the combination regimen was UST + VDZ in six cases (33.33%) and UST + TOF in five cases (27.78%). The DTT regimen was VDZ + ADA in two (11.11%) non-responsive CD patients, and both had a history of IFX failure. Up to the end time point, the duration of DTT treatment in 18 IBD patients ranged from 2 months to 51 months.

**Table 3:** Clinical data maintained on DTT.

Patients	Drugs of DTT	Months of DTT	Clinical response (month 3)	Clinical response (month 6)	Clinical response (month 9)	Endoscopic response	Still on DTT	AE
UC1	UST + TOF	8	Yes	Yes	NA	Yes	Yes	No
UC2	UST + VDZ	4	Yes	NA	NA	Yes	Yes	No
UC3	UST + TOF	51	Yes	Yes	Yes	Yes	Yes	No
UC5	UST + VDZ	2	NA	NA	NA	NA	Yes	No
CD1	VDZ + UST	11	Yes	Yes	Yes	Yes	Yes	*C. diff* infection
CD2	UST + TOF	8	Yes	Yes	NA	NA	Yes	No
CD3	IFX + UST	6	Yes	Yes	NA	Yes	Yes	No
CD4	UST + ADA	9	Yes	Yes	Yes	NA	Yes	Bacterial intestinal infection
CD5	UST + VDZ	11	Yes	Yes	Yes	NA	Yes	No
CD7	UST + VDZ	11	Yes	Yes	Yes	Yes	Yes	No
CD8	UST + VDZ	9	Yes	Yes	Yes	Yes	Yes	No
CD9	UST + VDZ	5	Yes	NA	NA	NA	Yes	No

NA, not applicable.

UC5 used DTT for 2 months.

**Table 4:** Clinical data discontinued DTT (monotherapy or converted to other drugs).

Patients	Drugs	Months of DTT	Clinical response (month 3)	Clinical response (month 6)	Endoscopic response	Months of monotherapy	AE
UC4	UST + TOF	5	Yes	NA	NA	1	Hair loss (UST-related)
UC6	UST + IFX	6	Yes	Yes	Yes	15	No
UC7	IFX + UST	6	Yes	Yes	NA	6	No
UC8	VDZ + TOF	4	Yes	NA	NA	1	Allergic purpura (TOF-related)
CD6	ADA + VDZ	4	No	NA	NA	other	No
CD10	ADA + VDZ	5	No	NA	No	other	No

NA, not applicable.

At the study endpoint, one patient remained on DTT for 2 months, two patients for 3 months, three patients for 6 months, and six patients for 9 months (Table 3), while six patients discontinued therapy (Table 4). Among those who discontinued, DTT was maintained for 4–6 months before cessation. Two UC patients with an endoscopic response transitioned to monotherapy with one of the combination agents. The remaining two UC patients switched to upadacitinib after discontinuing combination therapy due to persistent adverse effects.

Additionally, two CD patients failed to achieve a clinical response after more than 4 months of DTT and subsequently discontinued combination therapy. Follow-up of these patients revealed that one case transitioned to UST monotherapy due to changes in local health insurance policy. The second patient restarted AZA combination therapy at a reduced dose (12.5 mg/d instead of the previous 50 mg/d) due to comorbid ankylosing spondylitis and a history of herpes zoster with the higher dosage.

### Clinical, endoscopic, and laboratory responses to dual-target therapy

Among the patients who underwent DTT, the clinical response rates were 88.23% (15/17) at 3 months, 91.67% (11/12) at 6 months, and 100% (7/7) at 9 months. Endoscopic evaluation conducted on nine patients within the 9-month follow-up period revealed positive responses in eight patients, resulting in an endoscopic response rate of 88.89%.

After 6 months of DTT, UC patients (*n* = 4) showed a marked decrease in CRP levels (mean pre-treatment: 19.43, 6-month mean: 1.435) and increased albumin levels (mean pre-treatment: 40.95, 6-month mean: 43.5). For CD patients (*n* = 7) who underwent 6 months of DTT, our results demonstrated a notable reduction in CRP levels (mean pre-treatment: 11.93, 6-month mean: 7.16) and elevation of albumin levels (mean pre-treatment: 38.79, 6-month mean: 42.96). Five patients completed 9 months of DTT, showing a trend toward decreased CRP levels (mean pre-treatment: 13.16, 9-month mean: 6.38) and improved albumin levels (mean pre-treatment: 38.66, 9-month mean: 41.74).

### Adverse events and safety profile

While DTT demonstrated promising efficacy, several adverse events were observed during the treatment course that required clinical management. One patient with UC who received UST + TOF developed mild arthralgia and severe hair loss after the first UST application, and arthralgia persisted after the second subcutaneous injection while hair loss worsened. Arthralgia and hair loss improved significantly after discontinuing the UST application for 1 month. Another patient with UC treated with VDZ + TOF developed allergic purpura after starting these medications, which significantly improved after discontinuation of TOF. Both cases continued to receive single-drug maintenance therapy on an outpatient basis. In the third month of treatment, two CD patients developed infections: *Clostridium difficile* infection occurred in one patient following VDZ + UST therapy, and another bacterial intestinal infection developed in a second patient after ADA + UST therapy. They were treated with oral antibiotics on an outpatient basis and then maintained on DTT with the original regimen.

## Discussion

In this study, we investigated the safety and efficacy of dual-targeted therapy (DTT) in patients with refractory IBD. Recent exploratory studies on DTT have identified two distinct patient populations: (1) those with refractory IBD characterized by uncontrolled inflammation despite exhausting conventional treatment options and (2) patients with “double indication,” presenting with both IBD and extraintestinal manifestations (EIMs), where at least one condition remains active (9, 11). When considering DTT implementation, it is crucial to evaluate both biological safety profiles and mechanisms of action. The promising results from recent clinical trials support further exploration. The VEGA and EXPLORER studies demonstrated favorable outcomes regarding clinical remission and endoscopic response (17, 18). A recent Asian study reported 57.4% clinical remission and 51.7% endoscopic response with biologic agents and small molecules combination, but with simpler clinical response criteria (HBI reduction ≥3 points for CD or PRO2 reduction ≥50% for UC). Our research implemented stricter composite endpoints: For UC patients, we required a partial Mayo score reduction of ≥3 points with at least a 30% decrease from baseline plus a bleeding subscore reduction of ≥1 point or a bleeding subscore of ≤1 and for CD patients, beyond an HBI reduction of ≥3 points, those with HBI ≤ 4 needed to meet additional criteria of either CRP normalization or a Limberg score reduction of ≥2 points. This more rigorous endpoint design is clinically significant as it evaluates not only symptomatic improvement but also incorporates objective inflammatory markers and ultrasound score improvements, potentially reflecting disease activity more accurately and enhancing the reliability and clinical value of our findings. Additionally, we implemented rigorous therapeutic drug monitoring (TDM) for patients receiving infliximab and ustekinumab, leading to a 78.7% clinical response, confirming the efficacy of DTT in this challenging patient population (16).

Our results demonstrated that all six patients on corticosteroids at DTT initiation successfully discontinued them within the first 3 months. By the ninth month of treatment, we observed 100% clinical response and 88.89% endoscopic response, which aligned with results reported by Ribaldone et al. (13) and surpassed outcomes from some smaller case series (23, 24). Among four UC patients who transitioned to monotherapy, two switched due to adverse effects from one of the biologic agents yet maintained clinical remission during short-term (1-month) follow-up. The other two discontinued infliximab after 6 months of DTT while on ustekinumab, having achieved clinical, laboratory, and/or endoscopic remission. Notably, both patients maintained remission at their respective 6- and 15-month follow-ups, as evidenced by clinical parameters, laboratory markers (including fecal calprotectin), and endoscopic findings. These cases provide valuable clinical insights regarding the possibility of de-escalating from DTT to monotherapy after achieving disease control, potentially maintaining remission while reducing safety and financial concerns. Similar to strategies developed for transitioning from combination therapy with IFX (25), our findings contribute to the emerging evidence supporting carefully managed DTT de-escalation in select patients who achieve deep remission.

Monitoring treatment-related adverse events (AEs) remains equally important in clinical practice. Previous studies reported AE incidence rates ranging from 13 to 38.9% (11, 13, 23, 25, 26), including serious complications such as infections requiring hospitalization and skin cancer (23, 26). Notably, the EXPLORER study of triple combination therapy documented a drug-related AE incidence of 30.9%. Moreover, the VEGA study reported even higher side effect rates of 44% at 12 weeks and 63% at 50 weeks (17, 18). In our cohort, the AE incidence during DTT was 22.22% (4/18). Two patients with CD developed *C. difficile* and other bacterial intestinal infections in the third month of treatment but were able to continue their DTT regimen after outpatient management with oral antibiotics. Two UC patients experienced intolerable AEs leading to therapeutic adjustments—one developed allergic purpura while taking tofacitinib in the third month, while another experienced mild arthralgia and severe alopecia after ustekinumab administration in the fourth month. The hair loss gradually resolved after discontinuation, consistent with previously reported ustekinumab-related alopecia in the literature (27). Based on our observations, the 3–6 months period after DTT initiation appears critical for monitoring both treatment efficacy and potential adverse events. In contrast, a previous study reported the median time to adverse events during DTT as 5.1 ± 4.8 months, with severe AEs occurring at 4.1 ± 3.6 months (25). Notably, no serious complications requiring hospitalization or surgical intervention occurred. Screening for latent tuberculosis (TB) infection remains crucial to prevent TB reactivation (28). Neither our data nor existing studies have identified TB reactivation during DTT. These findings suggest that, while certain AEs may occur during DTT, they generally remain manageable within routine clinical monitoring parameters. Analyzing our data alongside previous studies indicates that AE frequency, particularly infections, increases slightly when TNF inhibitors are combined with other biologic agents or when small-molecule drugs are used with biologic agents (13, 25). Furthermore, the frequency of adverse effects increases markedly with the concomitant use of immunosuppressive agents or corticosteroids (25).

This study has limitations, including a small retrospective sample size and a relatively short follow-up duration. Another limitation is that our comparison of endoscopic improvement rates at 3, 6, and 9 months may introduce selection bias, as patients followed through to 9 months likely represent those with better treatment responses and tolerance. Additionally, comprehensive therapeutic drug monitoring (TDM) data were not available, as drug concentration testing in our setting incurs substantial out-of-pocket expenses and is typically reserved for patients with inadequate clinical response. Future prospective studies are necessary to better evaluate DTT’s long-term efficacy and safety while systematically incorporating TDM measurements. Nevertheless, our real-world data suggest that DTT is a promising option for refractory IBD patients, offering a relatively safe and effective path to remission.

## Data Availability

The original contributions presented in the study are included in the article/supplementary material, further inquiries can be directed to the corresponding authors.
